# The Application of Wearable Sensors and Machine Learning Algorithms in Rehabilitation Training: A Systematic Review

**DOI:** 10.3390/s23187667

**Published:** 2023-09-05

**Authors:** Suyao Wei, Zhihui Wu

**Affiliations:** 1 College of Furnishings and Industrial Design, Nanjing Forestry University, Nanjing 210037, China; 2 Co-Innovation Center of Efficient Processing and Utilization of Forest Resources, Nanjing Forestry University, Nanjing 210037, China

**Keywords:** wearable sensor, machine learning, disease rehabilitation, rehabilitation training

## Abstract

The integration of wearable sensor technology and machine learning algorithms has significantly transformed the field of intelligent medical rehabilitation. These innovative technologies enable the collection of valuable movement, muscle, or nerve data during the rehabilitation process, empowering medical professionals to evaluate patient recovery and predict disease development more efficiently. This systematic review aims to study the application of wearable sensor technology and machine learning algorithms in different disease rehabilitation training programs, obtain the best sensors and algorithms that meet different disease rehabilitation conditions, and provide ideas for future research and development. A total of 1490 studies were retrieved from two databases, the Web of Science and IEEE Xplore, and finally 32 articles were selected. In this review, the selected papers employ different wearable sensors and machine learning algorithms to address different disease rehabilitation problems. Our analysis focuses on the types of wearable sensors employed, the application of machine learning algorithms, and the approach to rehabilitation training for different medical conditions. It summarizes the usage of different sensors and compares different machine learning algorithms. It can be observed that the combination of these two technologies can optimize the disease rehabilitation process and provide more possibilities for future home rehabilitation scenarios. Finally, the present limitations and suggestions for future developments are presented in the study.

## 1. Introduction

With the rapid development of information technology, traditional medical rehabilitation methods combined with various disciplines and technologies, such as wearable sensors and machine learning algorithms, are widely used in clinical diagnosis, rehabilitation medicine, and other fields [1,2]. Cervical spine diseases, musculoskeletal diseases, stroke, cerebral palsy, hand paralysis, lower-limb paralysis, Parkinson’s, and other diseases require long rehabilitation periods. Wearable sensors and machine learning technology can assist clinicians in monitoring and predicting the prognosis and rehabilitation of patients. For example, Vijay placed the IMU (inertial measurement unit) on the chest and thighs of a patient to collect data on walking activities, such as standing, climbing stairs, cycling, etc., to complete the monitoring of the patient’s rehabilitation process [3]. Wearable sensors are an important technology for gait analysis, diagnosing walking disorders in patients with gait disorders, and gait analysis is very important for the clinical assessment of patient rehabilitation [4]. Patients with hemiparesis, such as apoplexy, usually must observe and evaluate hand-movement performance during the rehabilitation training period. Therefore, wearable sensors that do not affect limb movement can be worn for tracking and monitoring purposes. The feedback on joint movement information is crucial for the adjustment of and change in the rehabilitation treatment process [5]. Machine learning technology can integrate and predict the data obtained by sensors used for disease rehabilitation, thereby improving the accuracy of diagnoses of stroke and other diseases and assisting rehabilitation personnel in predicting the patient’s disease recovery trajectory [6,7,8].

Wearable sensors first appeared in the mid-20th century. As a hardware device, they can perform data interactions. According to different needs, users wear devices with specific functions to collect behavior or health records [9]. Wearable devices include a device body and sensor components, which are mechanically connected. They have different functions, principles, and forms, and are widely used in the fields of medicine and health [10]. Wearable sensors have the characteristics of convenience and a low price, providing researchers with a variety of possibilities and solutions [11]. Wearable sensors help rehabilitation patients to exercise at home, relieve travel pressure, and reduce psychological burden [12,13]. A variety of sensing devices are used to monitor patients’ vital signs and physiological responses, such as electromyography (EMG), electrocardiogram (ECG), and electroencephalogram (EEG), which can monitor the patient’s physical condition in real time. Electromyography (EMG) can determine the functional status of peripheral nerves, neurons, and muscles by receiving electrical activity signals when the muscles are at rest or contracting [14]. Electrocardiography (ECG) records the electrical activity of the heart by detecting the potential activity between cardiomyocytes and is commonly used to rapidly check for signs of arrhythmia [15]. An electroencephalogram (EEG) typically involves placing electrodes on a person’s scalp to detect changes in biological potential caused by brain activity. Brain waves contain a large amount of physiological and disease information. Through the processing of brain waves, doctors can perform the rehabilitation identification of patients’ brain diseases [16]. Gait analysis using wearable sensors, such as inertial sensors, gyroscopes, accelerometers, pressure sensors, etc., is widely used in many fields, such as neurorehabilitation and sports medicine. An inertial sensor is a sensor that detects and measures acceleration, tilt, vibration frequency, rotation angle, and multiple degrees of freedom (DOF) motion. They can convert motion signals into electrical signals, which are amplified and processed by electronic circuits [17]. A gyroscope is an angular motion-detection device that measures the angular velocity around multiple axes [18]. Accelerometers are sensors that measure changes in velocity in a single direction. Due to their low cost and strong reliability, they are often used in combination with various sensors [19]. A pressure sensor is generally composed of a pressure-sensitive element and a signal processing unit. It is a device that can sense the pressure on an object and convert the pressure signal into an electrical signal according to a certain rule. It is usually placed on the sole of the foot in gait recognition systems to obtain pressure information during movement [20].

Machine learning is a mechanism that uses computers to simulate human learning activities, enabling machines to learn autonomously without explicit programming, or researching how to effectively use information to obtain hidden and effective knowledge from big data [21]. Machine learning algorithms have been applied in different fields, such as finance, environmental protection, social media, and healthcare industries. In the medical field, machine learning is continuously upgraded and optimized in terms of disease analysis and data prediction [22,23,24]. With the advent of the era of big data, machine learning technology can efficiently acquire knowledge, conduct an in-depth analysis of complex and diverse data, and improve the accuracy of prediction results [25]. The commonly used algorithms of traditional machine learning mainly include the support vector machine algorithm (SVM), decision tree algorithm (DT), random forest algorithm (RF), artificial neural network algorithm (ANN), and so on. The support vector machine (SVM) algorithm is a supervised learning method that can be widely used in statistical classification and regression analysis [26]. Support vector machines are mainly used for face detection, image classification, and biological data mining. It is unlike the traditional way of thinking. It simplifies a problem by inputting the space and increasing the dimension, so that the problem can be reduced to a linearly separable classic problem [27]. The decision tree (DT) algorithm is an important classification and regression method in data mining technology, and its predictive analysis model is generally expressed in a tree structure [28]. The understandability of a decision tree model is affected by the size of the tree, its depth, and the number of nodes in the leaves. Decision tree has the characteristics of small levels of calculation and high accuracy [29]. The random forest algorithm (RF) integrates multiple trees through the idea of ensemble learning. The output category is determined by the mode of the output category of each tree and is mainly used for classification predictions [30]. This algorithm has the advantages of high precision, wide applicability, strong nonlinear data analysis ability, and overfitting difficulty [31]. The artificial neural network algorithm (ANN) is an algorithmic model that imitates the structure and function of biological neural networks [32]. Inspired by the neural organization of the human brain, the algorithm designs computing nodes similar to neurons and connects them to form a network. It transmits information rapidly and has strong generalization and nonlinear mapping abilities [33].

The review of wearable sensors and the machine learning algorithms in the literature mainly focuses on stroke rehabilitation [34], gait monitoring [35], fall prevention [36], and lower-limb movement [37,38]. For example, Jourdan et al. [39] focused on researching the application of commercial sensors, aiming at data collection of how sensors are applied, and seldom elaborated on the data processing that requires the application of machine learning technology. Usmani et al. [40] analyzed and compared the basic information of the participants, data sets, machine learning algorithms, sensor types, and where on the body they are worn and other parameters, and described the latest application of machine learning in fall monitoring and prevention systems. Boukhennoufa et al. [41] summarized the latest research progress in the field of stroke rehabilitation and compared the data processing of wearable sensors and machine learning algorithms.

At present, some reviews have summarized the latest research progress of wearable sensors and machine learning technology; however, a summary of disease rehabilitation training is lacking in the research. Many studies in the literature discuss the application of various sensors and machine learning techniques in the treatment and rehabilitation of certain diseases. For example, force sensors and bending sensors are added to stroke rehabilitation gloves to measure the grip strength and bending degree of the hand, and use machine learning technology to recognize gestures to promote the completion of the rehabilitation training process for patients [42]. Facciorusso et al. [43] used CiteSpace 6.1.R6 software to review the research status of sensor-based rehabilitation in neurological diseases, and to conduct a visual analysis of the research hotspots, authors, and journals. Yen et al. [34] reviewed the application trends of sensors in the remote monitoring and rehabilitation of neurological diseases, and discussed the functional evaluation elements that sensors should simulate. The abovementioned reviews are based on different perspectives of neurological diseases. According to the survey, there is no review summarizing the application and trend of the use of wearable sensors and ML technology in rehabilitation training for different diseases, which prevents researchers from making horizontal and vertical comparisons in this regard. Therefore, it is necessary to summarize the status of the use of wearable sensors and machine learning technology at present in different rehabilitation training scenarios for different kinds of diseases. The focus of the research should be on sensor location, sensor type, etc., as well as comparing the types and accuracy of machine learning algorithms to obtain the optimal algorithm. Sensors and machine learning-related information should be visualized to provide references for scholars to facilitate additional research. The research objectives of this review are as follows:

- It outlines the application of wearable sensors and machine learning technology in rehabilitation training;
- It specifically analyzes the sensor type, sensor location, and feature extraction applied in the recovery process of different diseases;
- It evaluates the type and accuracy of machine learning algorithms applied in different rehabilitation exercises;
- It discusses the limitations, trends, and directions of sensors and machine learning algorithms in rehabilitation applications.

The purpose of this study is to review the application of wearable sensor technology and machine learning algorithms in rehabilitation training for different diseases. The research results include the best sensors and ML algorithms that meet the rehabilitation conditions of different diseases, providing researchers with a choice of research directions and ideas for future research and development purposes.

## 2. Methods

This review used the Preferred Reporting Items for Systematic Reviews and Meta-Analyses (PRISMA) for the paper selection [44].

### 2.1. Search Method

The literature search used the Web of Science database and IEEE Xplore to retrieve all the literature published during the ten-year period from 1 January 2013 to 4 July 2023.

### 2.2. Document Retrieval

Firstly, the basic keywords used for the literature search were “wearable sensor”, “machine learning”, and “rehabilitation training”. Then, more relevant keywords were selected. The search formats of the two databases are shown in Table 1.

**Figure 1:**
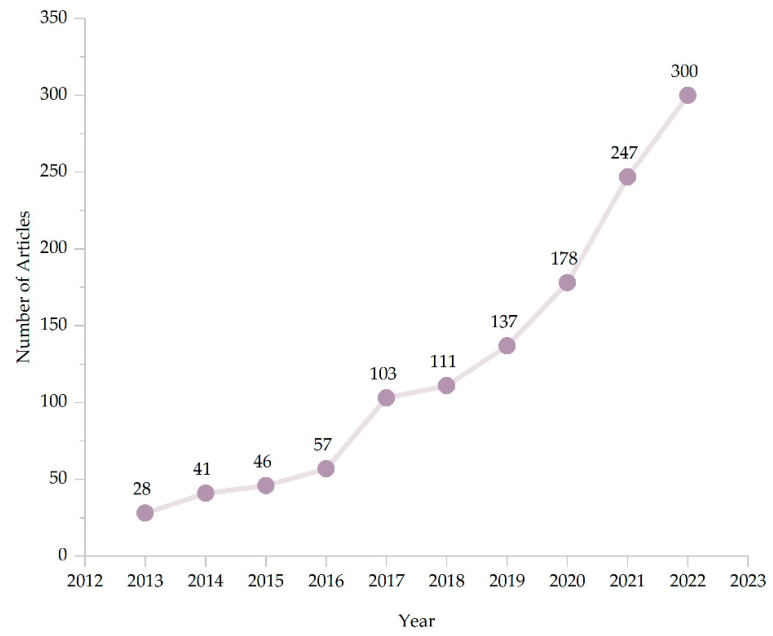
Number of relevant articles retrieved between the years 2013 and 2022.

Through the search, potentially relevant articles published between 1 January 2013 and 4 July 2023 were identified. Figure 1 presents the number of potentially relevant articles published per year between 1 January 2013 and 31 December 2022, after excluding duplicates. It can clearly be observed in Figure 1 that the number of published papers is clearly on the rise.

### 2.3. Screening Criteria

This review only included peer-reviewed journals or conference papers written in English between 1 January 2013 and 4 July 2023. The article research content was required to meet all of the following criteria:

1. The paper should include research conducted on wearable sensors, machine learning, and disease rehabilitation;
2. The paper should provide a detailed analysis of the performance characteristics, where the sensor is worn, and the accuracy of the wearable sensor;
3. The paper should elaborate on the application of the machine learning algorithm involved in data processing;
4. The paper should include research conducted on the treatment and rehabilitation of one or more diseases.

In addition, the abovementioned criteria should be followed for paper selection, the exclusion criteria were as follows:

1. Exclude all review papers, review articles, and papers that lack specific research results;
2. If the research exists in both academic journals and conference papers, select the former;
3. Exclude papers that briefly mention wearable sensors or machine learning or disease recovery.

### 2.4. Article Screening Process

The investigator (WSY) entered the data exported from the two databases into a table, which included information on the author, title, keywords, abstract, DOI number, etc. After excluding duplicate records according to the title and DOI number of the article, the investigator (WSY) then excluded the papers that did not meet the requirements according to the screening criteria based on the title, keywords, and abstract. Finally, the investigators (WSY and WZH) checked whether the specific content of the paper met all the screening requirements. The final screening was conducted and the selected papers were summarized.

## 3. Results

**Figure 2:**
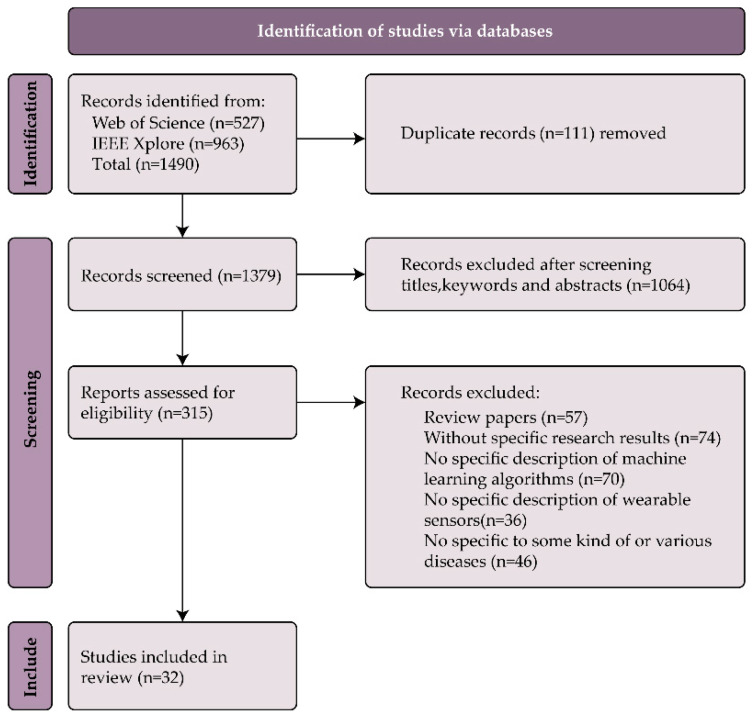
Literature screening process.

A total of 1490 documents were retrieved from the database, including 527 from Web of Science and 963 from IEEE Xplore. First, after removing 111 duplicates, 1379 papers were retained. Then, 1064 articles were excluded according to the title, keywords, and abstract. Then, 57 reviews were excluded. Subsequently, the full-text content was reviewed according to the screening criteria, 226 articles were excluded, and finally 32 articles were obtained. The whole process of document retrieval shown in Figure 2 was based on the screening results at each stage obtained by the method steps of PRISMA.

**Figure 3:**
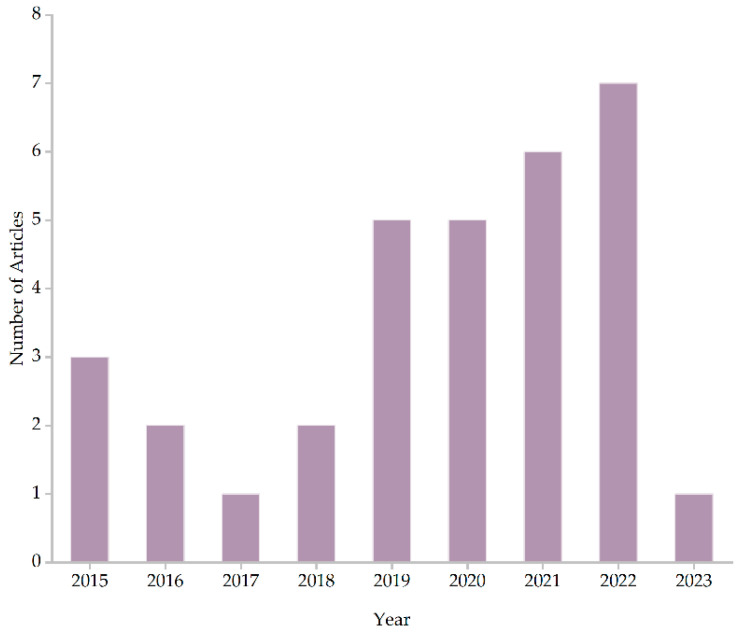
Year distribution of selected papers.

We sorted and summarized the 32 selected papers by year, and the results are shown in Figure 3. There were fewer papers in 2023 due to the deadline for the scope of the article search being 4 July 2023.

### 3.1. Wearable Sensors

Wearable sensors have the characteristics of light weight, flexibility, stability, and comfort. They are widely used for pulse and heartbeat monitoring purposes, gait analysis, and other health monitoring systems, disease diagnosis, and rehabilitation fields [45]. In the process of the diagnosis and rehabilitation of different diseases, different sensors are required to detect the physiological information required. In order to comprehensively evaluate the recovery of human health, it is sometimes necessary to work with multiple sensors. For example, a gait recognition system based on pressure and inertial sensors can obtain pressure information from the soles of the feet during exercise; inertial sensors can obtain dynamic information, such as acceleration and angular velocity, from different positions, such as on the thighs and ankles [20]. Based on the detected information, the recovery status of stroke patients can be evaluated and subsequent interventions can be performed.

Table 2 summarizes the specific results of the 32 screened documents on sensor type, wearable sensor location, sampling frequency, exercise, disease types, and other information.

### 3.2. Wearable Sensor Type

**Figure 4:**
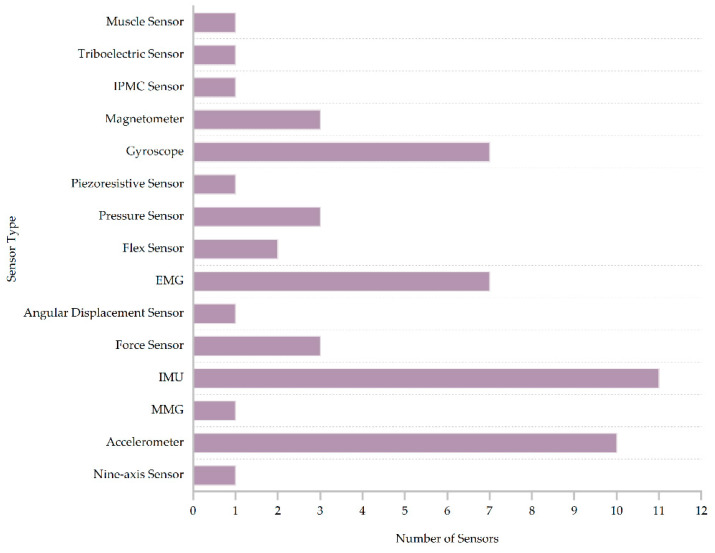
Quantity of each sensor.

Figure 4 summarizes the types of sensors used in the selected 32 papers. Muscle, IPMC, piezoresistive sensors, etc., have less applications and appear only once in all the articles. The top three applications were IMU, accelerometer, and EMG and gyroscope. Among them, IMU was the most widely used and appeared in 11 documents, accounting for more than 34%. An IMU is an inertial sensor composed of an accelerometer, gyroscope, and magnetometer. It can collect different types of data, such as acceleration and angular velocity values during motion to obtain more accurate motion measurement values. The researchers then evaluated and analyzed the motion processes based on the data [77]. An accelerometer presented the second highest number of applications and appeared in 10 studies. The accelerometer can detect the linear acceleration of the carrier and the direction of the acceleration, and it can be worn on the wrist to detect the activity of the arm. In the same way, when worn on the leg, it can detect the condition of the leg during walking or running movements, and the obtained data can reflect the use of the limb when playing sports [78]. The third most used sensor was the EMG and gyroscope, appearing in seven studies. EMG can be used to monitor and record myoelectric signals generated by skeletal muscle activity [79]. Gyroscopes can monitor angular velocity changes that occur during motion for motion posture analysis [80]. A force sensor, magnetometer, and pressure sensor are less frequently cited in the literature and were only mentioned in three papers. The flex sensor only appeared in two papers.

### 3.3. Disease Types

**Figure 5:**
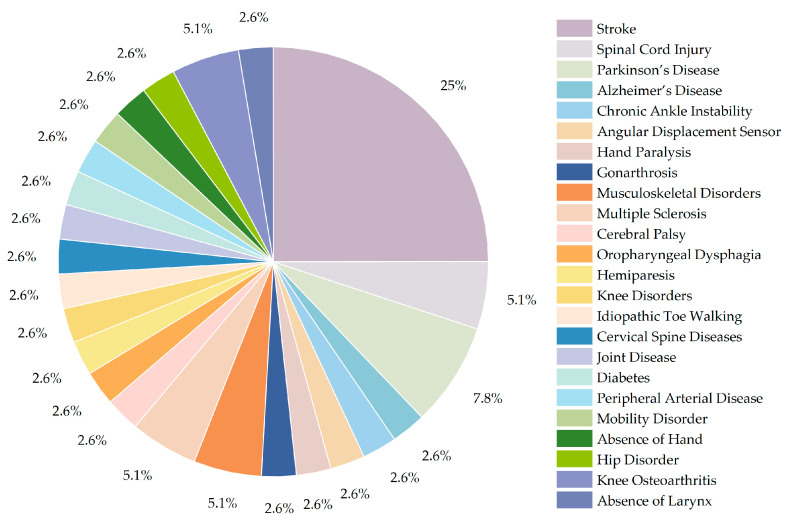
The proportion of various diseases.

Among the 32 selected papers we examined, a total of 24 diseases were addressed. As shown in Figure 5, the highest proportion is stroke, and 10 articles address this, accounting for more than 25%. This was followed by Parkinson’s disease, there are three articles that addressed rehabilitation treatment for this disease. Two articles were related to spinal cord injury, musculoskeletal disorder, multiple sclerosis, and knee osteoarthritis, respectively. The remaining 18 diseases, including chronic ankle instability, cerebral palsy, and knee disorders, were addressed by fewer studies, all of which only had one article published on the subject.

### 3.4. Sensor Location

**Figure 6:**
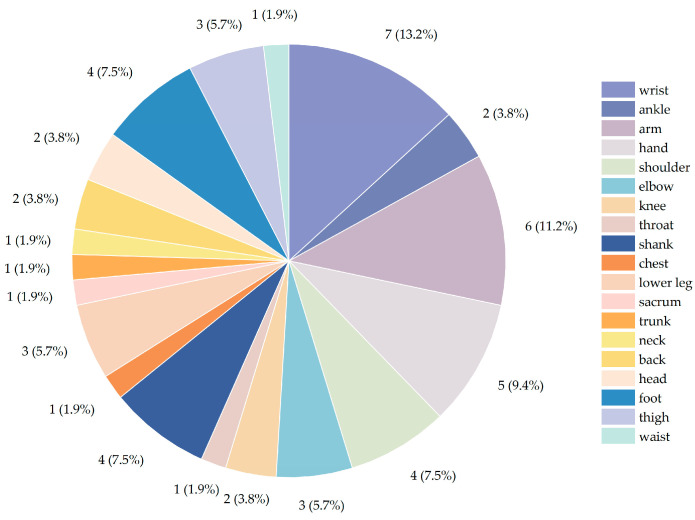
Locations where sensors are worn.

It can be observed in Table 2 that, among the locations where the sensors are worn, the wrists, arms, and legs are the body parts that are used the most frequently. It can be observed in Figure 6 that wearable sensors are mostly worn on the wrist, with seven articles mentioning this in the research. In second place is the arm, which is mentioned in six articles. Moreover, the hand is also one of the common placement areas for wearable sensors, and five articles were published on this. Then, four articles address sensors located on the shoulder, shank, and foot of the patient, respectively. Three articles mention the placement of the sensors on the elbow, thigh, and lower leg, and two articles concern the knee, back, and head. Only one article mentions the throat, chest, sacrum, trunk, neck, and left side of the waist. It can be observed from the results that wearable sensors are more commonly placed on the wrists, hands, legs, and shoulders; therefore, they are less frequently placed on the throat, chest, sacrum, trunk, neck, and waist. IMUs are most commonly used for monitoring the motion and acquiring the data of the wrist, leg, and arm so that motion in these areas can be quantified [81].

### 3.5. Rehabilitation Exercise

In the process of disease rehabilitation training, different training actions are adopted to achieve the effect of assisting the recovery of different diseases in patients. Rehabilitation training actions are determined by the type of disease and the site requiring rehabilitation. The analysis of the training movements in the rehabilitation process collected from the selected papers helps us to understand the research hotspots of disease rehabilitation. Among them, 19 articles addressed upper-limb movement, accounting for approximately 60% of the total; 13 articles addressed lower-limb movement, accounting for approximately 40%. The studies conducted on upper-limb movement mainly addressed the movements of hands (fingers, wrists, and palms), arms, shoulders, and other parts; the studies conducted on lower-limb movement primarily concerned the movements of the thighs, calves, and feet.

**Figure 7:**
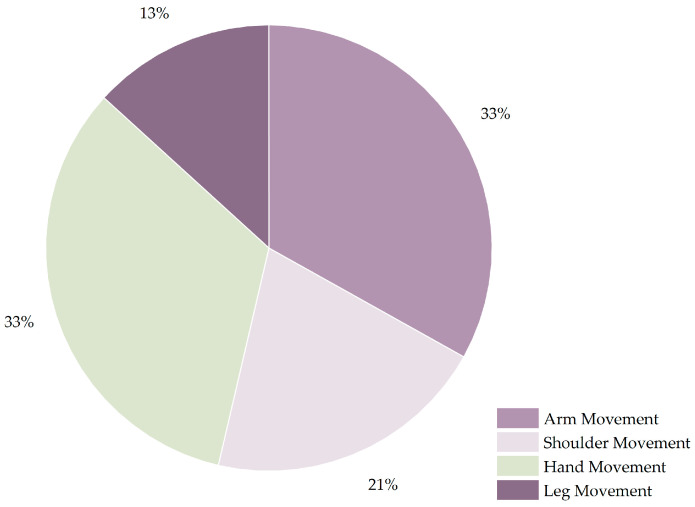
Rehabilitation movements for stroke.

Taking strokes as an example, different parts of the brain can cause different degrees of limb dysfunction, such as hemiplegia, impaired mobility, and a loss of hand function [82]. For information on rehabilitation training following a stroke, refer to Figure 7. Out of all the papers addressing stroke rehabilitation, hand and arm movements accounted for the 33 percent, which was the highest result, followed by shoulder movements at 20 percent and leg movements at 13 percent. It can be observed that the research performed on stroke rehabilitation, based on wearable sensors and machine learning technology, mainly focused on upper-limb movement; therefore, less research exists on lower-limb movement.

### 3.6. Feature Extraction

Feature engineering includes feature construction, extraction, and selection; the generation of features can be used as the input data for machine learning algorithms. Feature extraction is the optimization of a subset of features used to extract new features from the original features as the input [81]. The features are further divided into time-domain and frequency-domain features. Time-domain features can include data, such as the mean value, mean absolute deviation, root mean square, and peak value. The frequency-domain feature can contain data, such as the frequency band and total power between energy and entropy factors [47]. In this review, feature engineering was addressed and used in 23 of the selected articles. For different types of diseases, because of the different wearable sensors used and the different collected data types, the selection of features was also very different. In an article written on studying the walking gait of patients, the mean and standard deviation values of all the pressure data received by plantar pressure sensors were used as features [51]. There are also gait lines, regional pressures, gait phases, accelerations, step lengths, and joint angles that combine leg and plantar wearable sensor data as features [69]. In the remaining nine articles that did not mention or use feature engineering, the applied machine learning methods were neural network algorithms such as ANN, CNN, and NN. They did not require additional feature engineering. CNNs can self-learn and efficiently learn representative features obtained from large amounts of data by applying convolution operations to raw input data [71].

### 3.7. Machine Learning Methods

The machine learning algorithm is an application of artificial intelligence. It is based on data-trained algorithms that can automatically learn to continuously improve, make predictions, or act on data without being explicitly programmed [25,41]. Machine learning is divided into supervised learning and unsupervised learning behaviors. Supervised machine learning is a process of using samples of known categories to adjust the parameters of the classifier to achieve target performance. Unsupervised machine learning mainly involves discovering methods to solve various problems concerning pattern recognition from unknowns [83]. The field of application of machine learning is very broad. In the field of medical rehabilitation, the machine learning algorithm can predict the possibility of health outcomes by analyzing data and assist medical staff to take effective preventive measures [84].

Table 3 summarizes the relevant information about machine learning in the selected 32 papers, including the ML algorithm, accuracy, description, and limitation.

**Figure 8:**
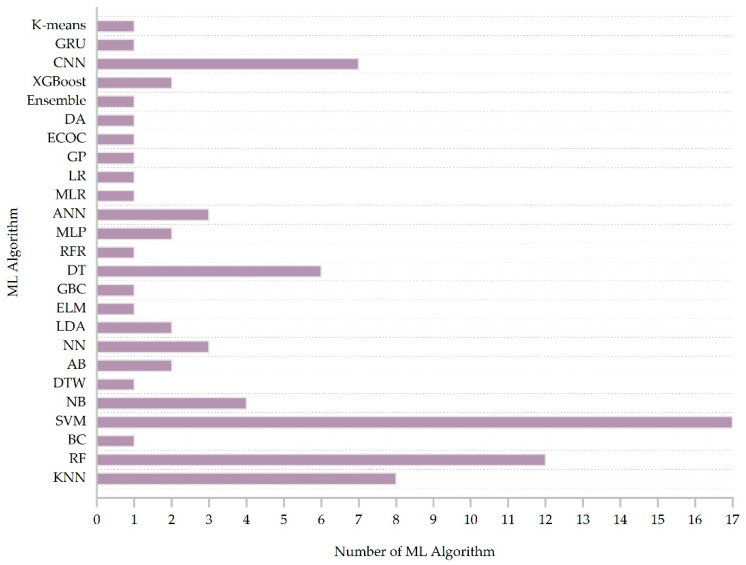
Number of different machine learning algorithms.

We summarized the machine learning algorithms that could be obtained from each article from the selected 32 documents and created statistics on all types of algorithms; the results are presented in Figure 8. The most widely used algorithm was SVM, which was used in 17 articles, accounting for more than 53% of the 32 articles. Followed by RF, 12 articles used this method. Then there was KNN, which was used in eight articles. Seven articles mentioned using the CNN method. Six articles mentioned using the DT method. Four articles mentioned using the NB method. ANN and NN, respectively were mentioned in three articles using this method. MLP, LDA, AB, and XGBoost were each mentioned in two articles using this method. The remaining 14 machine learning algorithms were only used in one document. From the abovementioned results, it can be concluded that SVM is favored by researchers. SVM has the characteristics of relatively easy training data and high accuracy; however, its shortcomings are also very obvious, such as slow learning speed and long training time [85].

## 4. Discussion

This systematic review included 32 papers based on wearable sensors and machine learning algorithms used to assess the degree of recovery of patients and assist rehabilitation training. On the one hand, this review summarized the relevant research results and determined that wearable sensors and machine learning algorithms can be better applied in the course of disease rehabilitation, helping doctors to keep abreast of patients’ recovery status and relieve social medical pressure. On the other hand, for the patients themselves, the application of wearable sensors facilitated their recovery at home, which could greatly reduce the factor of psychological burden. Therefore, it is necessary to summarize the application results of wearable sensors and machine learning algorithms in the field of disease rehabilitation, explore the limitations of the research, and propose the possibility of future studies.

We searched and screened papers for our analysis using the IEEE and the Web of Science core databases. The following section discusses (1) the selection of wearable sensor types in rehabilitation training; (2) the application of machine learning algorithms; (3) the analysis of the rehabilitation training process; and (4) the suggestions for future research.

### 4.1. Wearable Sensor Type Selection

This review determined that wearable sensors are more frequently used for upper-limb than lower-limb rehabilitation purposes. During the rehabilitation process of stroke patients, the recovery speed of the upper limbs was slower than that of the lower limbs. During the recovery process of the upper limbs, certain complications, such as shoulder pain, shoulder–hand syndrome, and upper-limb flexor spasms often occurred. Therefore, additional studies in the field are focusing on the upper-limb recovery of stroke patients [86]. He et al. [46] used a nine-axis sensor, including a three-axis accelerometer and a high-sensitivity three-axis gyroscope, in order to avoid the “drift phenomenon” caused by the lack of a magnetometer in the upper-limb rehabilitation evaluation of stroke patients. In this way, more accurate data can be obtained. If there is a long-term compensatory dependence on certain areas, such as the limbs and trunk, it affects the patient’s rehabilitation outcomes [87]. Xu et al. [49] used three different types of sensors, namely force sensor, angular displacement sensor, and sEMG, to realize the automatic detection of compensatory motion during the rehabilitation process of stroke patients. This method not only predicts the movement of the patient’s limbs, but also restricts the trunk from making relatively large compensatory movements, improving the safety and effectiveness of the patient during rehabilitation training. In a study performed on hand rehabilitation training for stroke patients, Yu et al. [50] used two acceleration sensors and seven bending sensors to monitor the motor functions of the arm, wrist, and fingers. This study comprehensively covered the upper limbs and provided a good understanding of the overall recovery of the upper limbs. For the rehabilitation of stroke patients’ fine hand movements, Chen et al. [42] used gloves integrating both force and flex sensors. Compared with gloves using biomedical signals, the gloves not only improved the signal quality, but also did not need to pay attention to the precision of electrode placement, thereby promoting the recovery of fine motor movements in stroke patients. Such hand rehabilitation systems can facilitate the development of IoT healthcare in the field of home rehabilitation. Kim et al. [54] considered patients in remote areas; therefore, they proposed in their study a wearable device equipped with a minimum number of IMUs to collect the characteristics of spastic movements, effectively improving the utilization rate of the device. Shradha et al. [61] improved the wearable device according to the use conditions of EMG, installed an IMU and EMG in the armband, and did not require the wearer to shave the hair in the area where the sensor is worn; therefore, it was more convenient to use. The device can also be designed in the form of a wristband. Biswas et al. [75] tracked the arm movements of stroke patients around the clock with a wristband inertial sensor to comprehensively assess the progress of rehabilitation. The research conducted on the rehabilitation of the lower limbs of stroke patients is often related to gait research, and gait research requires the cooperation of multiple sensors. Chen et al. [69] combined the plantar pressure sensor and IMU to obtain stable walking rehabilitation data through the combination of multi-directional data.

Parkinson’s disease is a common neurodegenerative disorder characterized by tremors, stiffness, and slowness of movement [88]. For the assessment of upper extremity symptoms in Parkinson’s disease patients, Huo et al. [48] designed the Parkinson’s diagnostic device (PDD) system, which can simultaneously assess three main symptoms. The PDD system is mainly composed of IMU and MMG sensors. Combining MMG signals can effectively improve the accuracy of symptom classification. For the gait research of Parkinson’s disease patients, Guo et al. [51] used plantar pressure sensors to efficiently collect patient’s plantar pressure data, and the selection of low-power sensors can effectively extend the daily monitoring time. From the perspective of users, Han et al. [57] selected a lighter IMU, which could reduce the patient’s exercise burden and ensure the completion of rehabilitation training.

It has become a trend in the research to apply wearable sensors for neck disease detection and rehabilitation purposes. For oropharyngeal dysphagia, Lee et al. [55] designed a self-powered IPMC sensor to detect throat muscle movements, which could more accurately identify actions such as coughing and swallowing. An [63] et al. designed a wearable neck device consisting of four silicone rubber triboelectric sensors and a silicone rubber collar. This device was highly flexibility, saved energy, and was cost-effective; therefore, it could be better used in the rehabilitation of neck diseases. Rameau [74] placed sEMGs on five joint muscles on one side of the face of laryngectomy volunteers who did not undergo radiotherapy. This method can realize silent speech recognition through surface muscle signals and help patients who have undergone laryngectomy and patients with impaired speech functions to perform speech rehabilitation techniques.

Most of the problems targeted by lower limb rehabilitation focus on lower limb dysfunction caused by spinal cord injuries, and diseases of the knee, hip, and other joints. In their study, Amir et al. [47] installed accelerometers on both the patient and assistive devices (crutches, wheelchairs, etc.). The information collected by the sensors placed on the assistive device presented a unique perspective, which combined the different perspectives of the patient and assistive device for the motion analysis. In order to relieve the pressure of patients with knee joint disease during the rehabilitation process, Antonio et al. [58] placed an IMU on the patient’s tibia to make the patient feel relaxed, and this was a labor-saving step employed during the rehabilitation training process. Moreover, Chen et al. [73] achieved the same effect by using a miniature inertial sensor with a lighter weight. Javier et al. [71] placed IMUs on the pelvis, thigh, calf, and foot of patients to collect different signals in the lower-limb gait study of hip joint disease rehabilitation training, so as to generate a comprehensive dataset for their analysis.

According to the statistical results of this review, 11 of the 32 papers used IMUs, which was the most frequently used sensor. IMU sensors not only have good wearability features and can be worn on any part of the wrist, arm, shoulder, and leg, but also collect kinematic parameters, such as body position, acceleration, and speed of motion with higher-accuracy results [89]. Therefore, they are favored by many researchers.

### 4.2. Application Analysis of Machine Learning Algorithms

In Section 3, it was observed that the data analysis of the machine learning algorithm in the process of disease rehabilitation can help predict the disease and help doctors and patients conduct more scientific and effective rehabilitation training techniques. The use of different machine learning algorithms enabled the comparison of analysis results based on the data set, the features extracted as input variables, and the complexity of the different models employed in the study.

A total of 17 papers used SVM to perform the disease rehabilitation evaluation, rehabilitation exercise classification, rehabilitation action recognition, and disease prediction steps. As a result of their high-accuracy characteristics, SVMs have a wide range of applications, and the number of SVMs used in the past decade in the field has been high compared to other ML algorithms [90]. He et al. [46] observed that the accuracy of SVMs was higher than that of k-nearest neighbor, RF, and Bayesian classifier algorithms for the upper-limb rehabilitation evaluation of stroke patients. It can be observed that the SVM is better than other machine learning classifiers for the classification of rehabilitation sports data. Due to the limited muscle strength available during a rehabilitation exercise, compensatory exercises inevitably occurred. Xu et al. [49] aimed at the detection of compensatory motion in rehabilitation exercises, and observed that the automatic compensation detection of SVM performed better than other algorithms in a normal rehabilitation exercise state. Chen et al. [34] combined features and used high-quality classification signals in their study, and observed that the average accuracy of the SVM algorithm was the highest. It is not difficult to observe that features such as input signals have a greater impact on the accuracy of machine learning algorithms. Lee et al. [55] proposed an optimized SVM algorithm based on SVM, which can produce high-accuracy results even when the sample size is small. This allows the SVM algorithm to cope with more diverse situations. Chen et al. [73] used a multi-layer support vector machine model capable of online segmentation, first through learning to extract the features that matched the target motion, and then accurately segment and classify the motion data online.

Deep learning methods also belong to machine learning methods, and their applications in the field of disease rehabilitation are gradually increasing in the field. CNN is an important neural network in the field of deep learning, which can be applied to many different scenarios and has an excellent learning ability [91]. Chae et al. [65] selected the CNN algorithm for home rehabilitation exercise detection. CNN has a high accuracy rate for human activity recognition, and does not require special feature extraction methods, and its classification is more streamlined than other algorithm steps. Zhu and Yen et al. [64,67] combined multiple CNN models and compared them with a single model and observed that the accuracy of the combined model was higher. It can be concluded that combining CNN models is a method that can effectively improve overall accuracy. An et al. [63] trained the model by adding data recorded under different conditions, which can also effectively improve the accuracy of the model. Guo et al. [51] applied the collected data features to a variety of machine learning algorithms, and the accuracy rates were generally similar; however, the accuracy of the neural network model was the highest, indicating that the amount of information collected by sensors can affect the accuracy of the model.

In addition to SVM and CNN algorithms, there are a variety of machine learning algorithms applied in the field of disease rehabilitation. Amir et al. [47] studied the two different perspectives of the patient and the mobility aid; therefore, algorithms, such as SVM, Bayesian, and DT, were used to detect physical activity results. We combined the Bayesian algorithm with joint classification algorithms, such as DTW, to detect activity patterns while using assistive devices. Rameau et al. [74] applied training data samples to different machine learning models, and then used the XGBoost model with the highest accuracy rate together with validation samples to create a predictive model for language recognition purposes. Although the abovementioned machine learning algorithms are rarely used in the research, their advantages are obvious under certain conditions. Therefore, no fixed machine learning algorithm is always better than other algorithms.

### 4.3. Rehabilitation

This review aimed to study the application of wearable sensors and machine learning algorithms in the field of disease rehabilitation. It was necessary to discuss the training required for various disease rehabilitation techniques.

According to the research, it can be observed that wearable sensors are most widely used in the rehabilitation of stroke diseases. The purpose of stroke rehabilitation training is to improve the patient’s ability to control their muscles, enhance the coordination of muscle groups, and improve the coordination ability for daily activities and body balance [92]. The most common symptoms of stroke are a limited movement of different parts of the body and gait disturbance. Patients require long-term intensive rehabilitation training to help them recover effectively [93]. Different scholars have conducted targeted research on different parts of the body of stroke patients with limited movement. For example, for the upper-limb rehabilitation of stroke patients, He et al. [46] used three movements: hand to lumbar spine, shoulder flexion, and forearm pronation. These three actions effectively covered the locations of all wearable sensors, which could help them accurately evaluate the rehabilitation of the upper limbs. Chen et al. [42] conducted research on the fine-grained training of hand rehabilitation for stroke patients. The purpose of the training was to improve the coordination functions of single and multiple fingers. During the rehabilitation training process, Kim et al. [54] arranged rehabilitation trainers to guide the patients to maintain correct movements and postures and improve the effect of rehabilitation training. Burns et al. [72] used a full-hand exoskeleton worn on the patient’s hand to assist the patient in grasping small items in daily life. Lower-extremity training after a stroke affects the future mobility of patients and is also of great importance. Chen et al. [69] provided visual feedback to patients during their rehabilitation training based on the gait characteristics collected by sensors, visualized lower-limb movements, stimulated patients’ awareness of gait correction autonomously, and effectively improved the quality of rehabilitation actions. In addition, Xu et al. [49] combined torso restraints with appropriate sensors for compensatory movements during the rehabilitation of stroke patients. The device effectively suppresses the compensatory movement that may occur during the rehabilitation training of the patient, and at the same time detects the movement trend of the patient during the training process to evaluate the accuracy of their rehabilitation actions.

Patients with spinal cord injuries must experience a long-term rehabilitation phase, which has a considerable impact on body motor functions [94]. Amir et al. [47] used various assistive mobility devices to improve the mobility of patients with spinal cord injuries while collecting information from the assistive devices and wearable sensors placed on the patient. The method provides ideas for helping researchers and healthcare professionals analyze the complex movements of patients during their rehabilitation. Guo et al. [51] aimed at the rehabilitation of Alzheimer’s, Parkinson’s, and other diseases, because the main rehabilitation training for such diseases lies in daily walking activity; therefore, a smart insole was used to monitor patients’ everyday walking activity. The design of such insoles has good development prospects for the rehabilitation of patients at home and in the community. Bavan et al. [53] applied five conventional rehabilitation movements for shoulder rehabilitation: shoulder abduction, shoulder flexion, wall sliding, wall pressing, and shoulder rotation. Among them, the four movements of shoulder abduction, shoulder flexion, wall sliding, and wall pressing were performed in a sitting position, the purpose of which was to reduce the compensatory movements of the other muscles during the rehabilitation process. Soangra et al. [60] focused on children’s idiopathic toe walking (ITW), reducing the size of the sensor and wearing it directly on the upper body. Not only did this not limit the walking rehabilitation movement, but it also helped parents monitor the child’s walking status in real time and presented abnormal gait occurrence. Javier et al. [71] used gait training as the basis for hip rehabilitation training, and strictly required patients to perform rehabilitation training once a day. For the rehabilitation of knee osteoarthritis, Enrica et al. [76] arranged rehabilitation training for different occasions, simulating both indoor and outdoor situations, to ensure the authenticity of the patient’s rehabilitation data.

### 4.4. Propositions for Future Studies

Based on the analysis of the selected articles, this review summarized some possible future development directions and some limitations of previous studies.

#### 4.4.1. Participants

In terms of the selection of experimental subjects, the experimental participants presented in some papers [48,50,53,54,56,57,58,60,65,69,70,71,73,74,75] selected disease patients or a combination of disease patients and healthy participants for the experimental research. Another approach [42,46,47,49,51,55,59,62,66,67,68,72,76] was to recruit disease-simulated subjects to imitate patients for exercise experiments. There was a certain gap between the information collected by simulated subjects and the real data of patients, and it was difficult to guarantee the authenticity and validity of the research results.

#### 4.4.2. Multiple Sensors and Special Patients

From the types of wearable sensors summarized in Table 2, it can be observed that the simultaneous use of multiple sensors has been a research trend in recent years [42,46,48,49,50,53,54,56,57,58,60,61,65,67,68,69,70,71,73,75,76]. However, sensors worn in different positions on the body pose a considerable challenge to data integration due to their different sampling frequencies. A variety of sensors can be combined to monitor the different movement trajectories of patients and comprehensively evaluate functional rehabilitation and daily-life activities At present, gait research systems based on pressure sensors are widely used in the field of rehabilitation training; however, insufficient attention has been paid to special foot-type rehabilitation research [4,95,96]. Therefore, in future research, special patients with diseases can be the key research objects. For example, in the gait research of Parkinson’s disease patients, a new rehabilitation training system can be established for patients with flat feet.

#### 4.4.3. Robot-Assisted Rehabilitation System

A robot-assisted rehabilitation system is an emerging intelligent rehabilitation training system in the field. The main function of the robot is to help patients train by simulating normal activities. It can also be worn on the patient to force them to perform various rehabilitation exercises, continuously stimulating their brains, improving the ability of their motor organs, and achieving an early recovery [97]. Robot-assisted rehabilitation systems mostly monitor hand extension, flexion, and wrist movements in the field of stroke rehabilitation, and there are few studies on fine movements, such as finger coordination activities.

#### 4.4.4. Sensor Durability

In the study of joint rehabilitation training such as those on the knee joint, wearing the sensor at the joint position affects the accuracy of motion detection during the movement of the joint [98,99]. The durability of the sensor is an issue worthy of consideration in the research [100]. The repeated bending of the flexible sensor leads to a decrease in durability of and damage to sensor function. Therefore, how to improve the durability of wearable sensors for long-term wear is also one of the focuses of the research in the future. In response to such problems, researchers have proposed that stretchable and flexible sensors can be placed in joints together to reduce the bending loss of flexible sensors installed in the joints [72]. Therefore, how to solve the durability problem of wearable sensors in other ways is also a focus of the research to be conducted in the future.

#### 4.4.5. Virtual Reality

The combination of VR and disease rehabilitation training has become a development trend in the field. From the perspective of interaction, increasingly more researchers have proposed that future rehabilitation training can use virtual scenes to improve the interest and autonomy of patients. At present, some studies in the literature combine VR and sensors to assist patients in effective exercise rehabilitation techniques [101,102,103]. Combining virtual reality gaming with a network of wearable sensors to monitor a patient’s recovery is a promising form of technology. However, the existing research is not comprehensive and more extensive and in-depth applications are necessary, and specific designs should be created to be applied to rehabilitation training for different diseases.

#### 4.4.6. Machine Learning Optimization and Deep Learning Methods

Machine learning, including deep learning, is developing very rapidly, and it involves a wide range of applications [104,105]. For machine learning in the field of disease rehabilitation, if a smaller data set is selected, the coverage is reduced and cannot be extended to more people; therefore, a greater amount of high-quality training data are needed [106]. Classification training cannot be generalized. For specific patients, a machine learning algorithm trained separately should be used for classification purposes, because this can affect the overall classification effect by adjusting a single specific feature space to maximize the recognition performance [59]. Some studies report that deep learning methods outperform classical machine learning algorithms. Model accuracy and generality can be improved by obtaining larger sample sizes and applying deep learning techniques [107]. Future research can focus on using novel machine learning techniques, such as CNNs, to bypass tedious steps, such as feature extraction calculations. 

## 5. Conclusions

This paper reviewed the research of wearable sensors and machine learning algorithms in disease rehabilitation training. It can be observed that using machine learning algorithms to process data obtained from wearable sensors is helpful for rehabilitation training for different diseases. Based on the results obtained by this review, it is concluded that IMUs are the most used sensors during rehabilitation. Most of the sensors used in disease rehabilitation are non-invasive, and the research on sensors in the field of disease rehabilitation should also pay more attention to other types of sensors. Machine learning algorithms such as SVM have a good auxiliary effect on data analysis and prediction in the process of disease recovery. In order to find the optimal solution, more algorithms should be used in experiments. In the future, other approaches can be tested to compensate for our deficiencies and complete a more comprehensive review of wearable sensors and machine learning algorithms in the field of medical rehabilitation. In the future, with the development of wearable sensor technology, characteristic data can be collected for additional diseases, so as to facilitate the understanding of the recovery status of diseases. At the same time, machine learning algorithms are transforming the field of healthcare. Smarter machine learning algorithms are being developed to help healthcare professionals improve diagnostic accuracy, predict the progression of a patient’s disease, and make personalized treatment recommendations. Combining the two methods increases the possibility of remote disease diagnosis and home rehabilitation, which may change the shortage of medical resources at present due to the aging population, to a certain extent. This review may not have included some relevant papers as the data were only collected from the Web of Science and IEEE Xplore. In addition, some recent high-quality papers may not have received enough citations.

## Figures and Tables

**Table 1:** Keyword strings used in database searches.

Academic Library	Search String
Web of Science	((TS = (wearable OR wearable sensor OR wearable device OR wearable sensing device OR accelerometer)) AND TS = (machine learning OR intelligent system OR deep learning OR SVM OR support vector machines OR random forest algorithms OR neural network algorithms OR multilayer perceptron OR artificial neural networks OR ANN)) AND TS = (rehabilitation OR recovery OR rehabilitation training)
IEEE Xplore	(“All Metadata”: wearable OR “All Metadata”: wearable sensor OR “All Metadata”: wearable device OR “All Metadata”: wearable sensing device OR “All Metadata”: accelerometer) AND (“All Metadata”: machine learning OR “All Metadata”: intelligent system OR “All Metadata”: deep learning OR “All Metadata”: SVM OR “All Metadata”: support vector machines OR “All Metadata”: random forest algorithms OR “All Metadata”: neural network algorithms OR “All Metadata”: multi-layer perceptron OR “All Metadata”: artificial neural networks OR “All Metadata”: ANN) AND (“All Metadata”: rehabilitation OR “All Metadata”: recovery OR “All Metadata”: rehabilitation training)

**Table 2:** Wearable sensors used for rehabilitation training in selected papers.

References	Wearable Sensors Type	Participants	Sensor Location	Feature	Sampling Rate	Exercise	Disease Type	Methods
[46]	Nine-axis sensor (non-invasive)	10 post-stroke hemiplegic-simulated subjects Male: 7 Female: 3	Wrist	Mean value/standard deviation/root square mean value of motion tasks	100 Hz	Hand to lumbar spine/shoulder flexion 90 degrees/forearm pronation	Post-stroke hemiplegia	Upper-limb evaluation method in the Fugl–Meyer scale
[47]	Accelerometer (non-invasive)	Two individuals without spinal cord injuries	Wrist/ankle	Time domain feature: mean/mean absolute deviation/peaks; frequency domain features: total power between a band of frequencies/energy/entropy	32 Hz	Wheelchair propulsion/walking/walking using crutches	Spinal cord injury	Framework that uses a combination of machine learning models and wearable sensors to capture and track assistive technology-based mobility and function in individuals with SCI
[48]	MMG/IMU/force sensor (non-invasive)	23 Parkinson’s disease patients Male: 12 Female: 11 10 healthy subjects Male: 8 Female: 2	Upper arm/forearm/wrist/hand	Rigidity features: mean and standard deviation of the calculated torque/standard deviation of the joint angle and angular velocities, etc. Bradykinesia features: root mean square of pronation/supination motion speeds, etc. Tremor features: means and standard deviations of processed rates-of-turn and accelerations	100 Hz	Pronation supination movements	Parkinson’s disease	Establish a new PDD model and evaluate it using Unified Parkinson’s Disease Rating Scale scores
[49]	Force sensor/angular displacement sensor/sEMG (non-invasive)	Fifteen healthy, right-handed male subjects aged between 22 and 30 years old	Hinge mechanism/trapezius muscle	RMS of the force sensor/RMS of the angular displacement sensor	-	Side-to-side reaching/back and forth/ up and down	Stroke	By combining force, angular displacement, and electromyographic signals with torso constraints as the main body, automatic detection of compensated motion is achieved
[50]	Accelerometer/flex sensor (non-invasive)	24 stroke patients Male: 16 Female: 8	Shoulder/elbow/wrist/fingers	AMP /MEAN /RMS/JERK/ApEn	20 Hz	Shoulder anteflexion/shoulder extension/forearm pronation and supination/lumbar touch/wrist flexion and extension/lateral pinch/finger touch	Stroke	A novel remote quantitative Fugl- Meyer evaluation (FMA) framework that maps sensor data to clinical FMA scores
[51]	Pressure sensor (non-invasive)	13 young participants Male: 7 Female: 6	Plantar	Means and standard deviations of all the pressure data	100 Hz	Standing/walking/siting	Alzheimer’s disease/Parkinson’s disease/chronic ankle instability	Long-term center of pressure monitoring system in a smart-shoe form
[42]	Force sensor/flex sensor (non-invasive)	8 subjects with normal hand motor functions Male: 5 Female: 3	Knuckle/fingertips/palm	MAV/ RMS/ WL/VAR /standard deviation	200 Hz	Finger flexion	Hand paralysis	Hand rehabilitation system that supports both mirror therapy and task-oriented therapy
[52]	Piezoresistive sensor (non-invasive)	18 healthy subjects Male: 9 Female: 9	Knee	-	18.75 Hz	Open-chain knee flexion	Gonarthrosis	Instrumented knee sleeve and modeled using an adaptive enhanced RFR model
[53]	Accelerometer/gyroscope/magnetometer (non-invasive)	20 patients Male: 8 Female: 12	Shoulder	Time domain features: mean/root mean square/standard deviation, etc. Frequency domain features: maximum frequency component/mean frequency component/energy spectral density, etc.	100 Hz (accelerometer) 100 Hz (gyroscope) 25 Hz (magnetometer)	Shoulder abduction/shoulder flexion/wall slide/wall press/shoulder rotation	Musculoskeletal disorders	Using a single inertial sensor and supervised machine learning technology to identify and classify shoulder rehabilitation activities
[54]	Accelerometer/gyroscope/magnetometer (non-invasive)	48 patients Male: 26 Female: 22	Dorsal side of the elbow	Root mean square/mean/standard deviation/energy/spectral energy/absolute difference/variance/SMA/SV	256 Hz	Elbow flexion and extension movements	Stroke/multiple sclerosis/cerebral palsy/spinal cord injury	Machine learning algorithms and inertia signals collected during passive stretching are used to grade spasms
[55]	IPMC sensor (non-invasive)	-	Throat	Raw voltage data	-	Cough/hum/nod/swallow	Oropharyngeal dysphagia	Self-powered IPMC sensor that can distinguish between the different pressures exerted by throat movements
[56]	IMU (non-invasive)	10 healthy and 12 post-stroke volunteers	Fingertip/hand	Mean value of movement intensity/smoothness of MI/average acceleration and rotation energy, etc.	100 Hz	Arm movements	Hemiparesis	IMUs used to recognize the purposeful and non-purposeful movements in ADLs for identifying and promoting the use of the impaired limb during daily life in people affected by stroke
[57]	IMU (non-invasive)	25 PD patients and 28 healthy subjects	Ankle/shank	SL/GD/PSP/MH/RL/RSZ/RSY/RSX/MPV/MVV/MSV/MHD	100 Hz	Walk	Parkinson’s disease	Novel method for automatic assessment of the gait task in UPDRS based on only two shank-mounted IMUs and 12 m straight walking test
[58]	IMU/accelerometer/gyroscope (non-invasive)	44 clinical and 10 healthy subjects	Shin	Mean/median/standard deviation/variance, etc.	102.4 Hz	Heel slide/seated knee extension/inner range quadriceps/straight leg raise	Knee disorders	System that provides patients with automatic feedback on knee rehabilitation exercises
[59]	EMG (non-invasive)	4 healthy male subjects	Lower leg	EMG data	-	Walk	Stroke/multiple sclerosis	New approach for spastic detection in hemiplegia-affected EMG data using the IPANEMA BSN in combination with SVM
[60]	Accelerometer /gyroscope (noninvasive)	36 pediatric patients	Trunk/sacrum/shank	Mean frequency/the first 5 DFT coefficients/the first 5 maxima of DFT coefficients and their corresponding frequencies	75 Hz	Walk	Idiopathic toe walking	Using wearable sensors and ML, real-time step detection can be combined with assistive devices for intervention and motor rehabilitation purposes
[61]	IMU/EMG (non-invasive)	-	Arm	Mean absolute value/standard deviation/variance/root mean square/waveform length/zero crossing/integrated EMG	50 Hz (IMU) 200 Hz (EMG)	Thumb and index finger movements	Stroke	Pattern recognition of thumb and index finger gestures using EMG signal recording obtained from Myo armband
[62]	EMG (non-invasive)	22 subjects	Forearm	Mean/variance of EMG/MAV, etc.	1000 Hz	Hand movement	Musculoskeletal disorders or injuries	An off-line classification approach for the 26 upper-limb ADLs included in the KIN-MUS UJI dataset
[63]	Triboelectric sensor (non-invasive)	-	Neck	-	-	Neck movement	Cervical spine diseases	A neck motion detector comprising a self-powered triboelectric sensor set and a deep learning module to recognize neck motion
[64]	Accelerometer (non-invasive)	49 healthy volunteers	Shoulders/back/elbows/forehead	-	32 Hz	Place hands behind the head with ten fingers crossed/push the elbows back to the body/stretch both hands up with ten fingers crossed/bend over to the left/right	Joint disease	Multi-path convolutional neural network (MP-CNN) based on sensor data for rehabilitation training recognition
[65]	IMU/accelerometer/gyroscope (non-invasive)	17 participants in the HBR group and 6 participants in the control group	Wrist	-	10 Hz	Bilateral shoulder flexion with both hands interlocked/wall push /move the scapula /towel slide	Chronic stroke	Home-based rehabilitation (HBR) system that identifies and records the type and frequency of rehabilitation exercises performed by the user
[66]	Pressure sensor (non-invasive)	12 healthy subjects	Foot	-	300 Hz	Walk	Diabetes/peripheral arterial disease	Method based on the artificial neural network to classify walking speed and walking time by using plantar pressure images
[67]	Accelerometer /gyroscope (non-invasive)	21 healthy male volunteers	Waist	-	50 Hz	Walk/walk upstairs/walk downstairs/sit/stand/lay	Mobility disorder	Device consisting of a single-board computer (SBC) and a six-axis sensor that recognizes activities through deep learning algorithm
[68]	EMG/muscle sensor (non-invasive)	5 healthy male subjects.	Arm	-	-	Hand open/hand close/pinch/pointing finger	Stroke/absence of hand	Method for controlling a 3D prosthetic hand using electromyographic data of basic gestures and manipulating the prosthetic hand using classified data
[69]	IMU/pressure sensor (non-invasive)	20 hemiplegic patients and 10 healthy individuals	Bilateral feet/bilateral calves/bilateral thighs/waist	Gait line/regional pressure/gait phase/acceleration/step length/joint angle	200 Hz	Walk	Stroke	Method for interpretable BRS-L evaluation of lower extremity motion data and plantar pressure data collected using IMUs and pressure sensors
[70]	IMU (non-invasive)	12 stroke patients Male: 7 Female: 5	Wrist	Mean of the signal/variance of the signal/ RMS, etc.	20 Hz	Arm movement	Stroke	Arm rehabilitation monitor system using an IMU sensor placed on a single wrist to acquire arm motion information and process the data using a machine learning classifier
[71]	IMU (non-invasive)	12 patients with hip unilateral arthroplasty	Foot/lower leg/upper leg/lower back	-	60 Hz	Walk	Hip disorder	Method used to monitor the progress of rehabilitation using kinematic data obtained from a wearable sensor system and a deep convolutional neural network
[72]	EMG (non-invasive)	5 healthy subjects Male: 4 Female: 1	Hand/arm	-	13.33 Hz	Wrist/elbow/shoulder flexions	Stroke	Flexible cable-driven full-hand exoskeleton to aid the rehabilitation of stroke patients
[73]	IMU (non-invasive)	10 subjects Male: 5 Female: 5	Chest/thigh (close to the knee)/shank (close to ankle) of the working leg	Angle of shank for SAE and QSM/angle of thigh for SLR	-	Short-arc exercise (SAE)/straight leg raise (SLR)/quadriceps strengthening mini-squats (QSM)	Knee osteoarthritis	Online segmentation method for knee OA rehabilitation monitoring that can provide real-time feedback to patients and physical therapists
[74]	sEMG (non-invasive)	Laryngectomee volunteer	Articulatory muscles on hemiface	Vector of all 0 values, except for 1 in elements where the target sEMG feature is represented	250 Hz	Specific facial expressions/palpating face	Absence of larynx	Method used for applying the machine learning algorithm to electromyographic signals of joint muscles to identify silent speech in patients undergoing a total laryngectomy
[75]	Accelerometer/gyroscope/magnetometer (non-invasive)	4 healthy subjects and 4 stroke patients	Wrist/arm	Standard deviation/ RMS/ information entropy, etc.	50 Hz	Extension and flexion of the forearm/rotation of the forearm about the elbow/rotation of the wrist about long axis of forearm	Stroke	Method using data collected from a wristband, a wireless three-axis accelerometer, and a three-axis rate gyroscope combined with partial k-means clustering to identify basic movements of the upper body in everyday life
[76]	IMU (non-invasive)	12 healthy subjects with no reported knee pain	Right knee	MDF/power of the spectrum/peak frequency/maximum spectral amplitude/output range of the signal in the time domain	122 Hz	Walk/run both indoors and outdoors/ travel up and down the stairs	Knee osteoarthritis	Sensor system capable of monitoring knee motion and classifying aspects of daily living activities to aid in the rehabilitation of patients with knee OA

Abbreviations used in table: MMG (mechanomyography), IMU (inertial measurement unit), EMG (electromyography), sEMG (surface electromyography), IPMC (ion-exchange polymer metal composite), IMMU (inertial and magnetic measurement unit), RMS (root mean square), SMA (signal magnitude area), SV (signal vector magnitude), MI (movement intensity), SL (stride length), GD (gait cycle duration), PSP (percentage swing phase), MH (max ankle height), RL (range of lateral displacement), RSZ (range of shank Z-axis rotation), RSY (range of shank Y-axis rotation), RSX (range of shank X-axis rotation), MPV (maximum progressive ankle), MVV (maximum ankle vertical velocity), MSV (maximum shank Z-axis angular), MHD (ankle displacement at MH), DFT (discrete Fourier transform), AMP (amplitude of sensor data), MEAN (mean value of sensor data), RMS (root mean square value of sensor data), JERK (root mean square value of the derivative of sensor data), ApEn (approximate entropy of sensor data), MAV (mean absolute value), RMS (root mean square), WL (waveform length), VAR (variance).

**Table 3:** Machine learning algorithms in the selected papers.

References	ML Algorithm	Accuracy	Description	Limitation
[46]	KNN/RF/BC/SVM	84.95% (KNN) 88.12% (RF) 85.05% (BC) **97.79% (SVM)**	The five-fold cross-validation method was used to divide the feature data and action labels into five groups, four groups were used to train, and the remaining group was used to validate the accuracy.	It can recognize upper-limb movements. It cannot identify lower-limb movements.
[47]	SVM/RF/NB/DTW	87.4% to 97.6%	Classification accuracy was assessed using multiple assessments, including 10-fold-stratified cross-validation and 50% cross-validation (50% for training, 50% for testing).	Lack of evaluation of a high number of individuals with varying degrees of spinal cord injuries.
[48]	KNN/AB/NN/RF	**85.1% (KNN(K = 1))** 83.0% (AB) 81.9% (NN) 73.6% (KNN(K = 3)) 72.4% (RF)	A voting classification model was established by combining three basic classifiers, and a soft voting algorithm was used to select the final UPDRS score.	A larger dataset needs to be established to reduce errors and improve the accuracy of the model.
[49]	KNN/SVM/LDA	**97.58 ± 3% (SVM)** 95.68% (KNN) 92.38% (LDA)	The nine extracted features were supplied to the LDA, KNN, and SVM. A five-fold cross-validation method divided the feature data and action labels into five equal groups. Four groups were used to train the classifiers and the other group was used to verify the accuracy of the classifiers.	It Is necessary to conduct actual clinical trials on patients to further verify the universality of detection equipment and prediction methods in identifying the abnormal movement patterns of patients.
[50]	ELM	-	Five characteristics were extracted for each exercise. Each exercise had 240 data samples, of which 200 samples served as the training set and the remaining 40 samples served as the test set.	The ceiling effect makes it difficult for doctors to accurately assess the patient’s motor functions.
[51]	SVM/RF/GBC/NN	97.9% (SVM) 97.9% (RF) 97.2% (GBC) **98.6% (NN)**	The input features were the means and standard deviations of the pressure data of the sensor, and then the features were transferred to the multi-class support vector machine (SVM) with the radial basis function (RBF) core as the classifier.	Only simple-activity testing was conducted, lacking complex-activity detection.
[42]	KNN/SVM/DT	**99.65% (SVM)** 96.27% (KNN) 81.73% (DT)	The optimal feature subset was selected from the original features and each feature was tested independently to evaluate the combination of different features using the 10 × 10 cross-validation.	Similar actions are easily mistaken.
[52]	RFR	-	Each model trains 90% of the data and tests the remaining 10% of the data. The hyperparameters of the multivariate machine learning regression were optimized using grid search and multivariate Bayesian optimization methods.	It is not possible to fully capture the peaks and troughs of all knee joint flexions or the magnitude of internal/external rotational degrees of freedom.
[53]	DT/SVM/KNN/RF	90.9% (DT) 95.7% (KNN) **97.2% (SVM)** 96.4% (RF)	Training used a subset of high-level features; two different validation methods were used to evaluate the prediction performance. Ten-fold cross-validation distributed all labeled data segments randomly and evenly across ten sections. The data contained in the nine folds were trained and the remaining data were tested.	Some actions are misclassified as junk activities, and similar activities are easily confused.
[54]	DT/RF/SVM/LDA/MLP	76.6% (DT) **91.8% (RF)** 71.8% (SVM) 80.6% (LDA) 82.6% (MLP)	The performance of the classifier was tested by leave-one cross-validation. Each classifier was tested under four different conditions to determine an optimal classifier.	Due to the limited sample size, it is not guaranteed to perform well on larger datasets.
[55]	SVM	95.0%	The training data set with the kernel function was used to train the SVM model, and the test data set was input into the model to check the accuracy. The model was optimized by punishing parameter C and gamma parameter g, which could test the probability of misclassifications.	When the cough is not strong enough, it is impossible to measure the amplitude in the signal, which can lead to an incorrect judgment.
[56]	SVM/ANN	81.20% (SVM) **97.06% (ANN)**	Purposeful events were randomly selected to evaluate the generalization ability of the machine learning model, and then the classifier was trained using all the parameters. The ten-fold cross-validation method was used to train and test the data.	There is a lack of data on other fingers and an age mismatch among participants in this study.
[57]	SVM/NBC/MLR	**73.6% (SVM)** **73.6% (NBC)** 66.0% (MLR)	Recursive feature elimination was performed on each model to study the relationship between the number of features and the accuracy, and to find the optimal feature selection.	The evaluation and data collection are not synchronized, which may lead to errors. The dataset is small and unevenly distributed, making it prone to overfitting and resulting in errors. The selection of gait features is not comprehensive.
[58]	LR/SVM/AB/RF/DT	SKE:86.05% (LR) **96.70% (SVM)** 94.13% (AB) 93.11% (RF) 91.75% (DT)	Each model was evaluated through a five-step cross-validation process. To avoid overfitting, the folds were generated by dividing the date set by the patient. In the classification process, the data set contained only duplicates that were correctly segmented.	Partial actions obtained the less satisfying results in the laboratory dataset.
[59]	SVM	-	The set of training vectors was created based on EMG signal data collected from two different patients, and then an SVM was trained, and the resulting structure could be stored by significant settings.	The results cannot represent all individual diseases.
[60]	SVM/DT/RF/KNN/MLP/GP	85.8% (SVM) 74.4% (DT) 82.8% (RF) **92.9% (KNN)** 85.8% (MLP) 86.8% (GP)	The data were randomly divided into two parts, training and testing, with a ratio of four to one. The data were normalized to between 0 and 1 using min–max scaling. Five cross-validations were used for each classifier’s training dataset.	More datasets are needed to achieve a better classification performance.
[61]	SVM/KNN/NB/ECOC/DA/DT/ensemble	**88.42% (SVM)** 80.09% (KNN) 73.04% (NB) 84.34% (ECOC) 81.73% (DA) 82.60% (DT) 85.65% (Ensemble)	The ratio of training to testing was 4:1, and the test set accuracy was displayed as the average accuracy of 10 trials. In order to achieve the best result, the linear kernel function was used.	The placement position of the armband has a significant impact on signal recognition.
[62]	SVM/RF/XGBoost/CNN/GRU	65.4% (SVM) 57% (RF) 47.7% (XGBoost) **83.6% (CNN)** 79% (GRU)	The classifier was trained and tested using TD and FD features. The integrated approach was built with the four models with the best training performance to evaluate methods that could improve the performance of individual models.	Similar movements with both hands can easily lead to confusion.
[63]	CNN	**92.63%**	The leave-one session-out (LOSO) policy was adopted. The data obtained from one session were used as the test dataset, and the data collected from the remaining three sessions were used as the training dataset. This procedure was repeated four times until the data for each session were considered as one test dataset.	-
[64]	MP-CNN	**90.63%**	Depending on the number of layers in the middle path, the correlation of the output of the last pooling layer was captured, and the accuracy was highest when D-CNN and S-CNN were combined.	More action data needs to be collected.
[65]	CNN	85.6~100%	Cross-validation was performed on different input and sensor data, and the model with the most accurate data was determined.	There is some degree of data loss.
[66]	ANN	**94%**	Flatten layer was used to convert the image of the plantar region into a one-dimensional value sequence. The sequence was then used as input data for the ANN model. Hidden layers to propagate training mechanisms.	The data set used is not comprehensive and the plantar features are not detailed.
[67]	CNN	**97.49%**	A feature fusion model containing nuclei of different sizes was used. After signal normalization and conversion into a fixed format, the inertial data were divided into three partitions, which were composed of three convolution layers and one flattened layer, respectively.	When the data characteristics of two actions are similar, classification errors are prone to occur.
[68]	ANN	**91%**	For ANN training and testing, a 3:1 ratio was used. The training and verification errors were reduced in a certain number of iterations.	No wrist motion and no force control.
[69]	RF/KNN/SVM/NB	80.07% (RF) **94.20% (KNN)** 75.35% (SVM) 82.43% (NB)	A cross-validation approach was used to evaluate the predictive performance of the classification model. The leave-one-subject-out strategy was used to divide the data into training and test sets.	-
[70]	RF/CNN	Home-Home: **77.1% (RF)** 76.6% (CNN)	A validation dataset was generated by separating 20% of the continuous portion of the training dataset obtained from each participant in a random location. The results of the validation dataset were used to tune the classifier hyperparameters.	Datasets are small and unrepresentative.
[71]	DCNN	**98%**	Training, validation, and test data were randomly divided into 70%, 15%, and 15%, respectively. The adaptive moment estimation method was used for optimization. The stop-loss criterion was applied to the training progress by evaluating the validation loss.	Lack of more detailed analysis of DCNN input data and gait kinematics data during rehabilitation process.
[72]	NN	-	The Bayesian regularization algorithm was used to train the neural network to minimize the internal parameters and model errors and avoid overfitting.	The data set is small and the system is not a fully closed loop.
[73]	SVM	90.6% (layer 1) **92.7% (layer 2)**	10x cross-validation was used to validate the data. A total of 10 rounds were performed, with 1 subset of the 10 subjects selected for each round as the training data and the other 9 subsets as the test data.	Patient movements cannot be fully simulated, and the data are not accurate enough.
[74]	XGBoost	**86.4%**	The feature data consisted of vectors representing all zeros in the elements that characterized the target surface EMG signal.	Need to improve silent speech recognition algorithm to realize the translation of silent speech into personalized synthetic speech.
[75]	K-means	HS: **88% (DOA)** 83% (DOG) SP: 70% (DOA) 66% (DOG)	The clustering was formed using a sorted list of features; therefore, a combination of 2–30 features was selected in turn in each iteration, and 10-fold cross-validations were performed on the selected feature vector ten times.	The effects of sensor fusion and other attachment positions need to be observed in larger sample populations.
[76]	RF	**93%**	The random forest algorithm was a collection of 10 classification decision trees, with 90% of the data randomly selected for building the tree and 10% for testing the algorithm.	Test data should include other activities of daily living to allow for a more comprehensive classification of activities of daily living.

Abbreviations used in the table: KNN (K-nearest neighbors), RF (random forest), BC (Bayesian classifier), SVM (support vector machine), DTW (dynamic time warping), NB (naive Bayes), LDA (linear discriminant analysis), ELM (extreme learning machine), GBC (gradient boosting classifier), DT (decision tree), RFR (random forest regressors), MLP (multilayer perceptron), ANN (artificial neural network), MLR (multiple linear regression), LR (logistic regression), AB (adaptive boosting), SKE (seated knee extension), GP (gaussian process), ECOCs (error correcting output codes), DA (discriminant analysis), MP-CNN (multipath convolutional neural network), CNN (convolutional neural network), DCNN (deep convolutional neural network), HS (healthy subject), DOA (data of accelerometer), DOG (data of gyroscope), SP (stroke patients), XGBoost (extreme gradient boosting), UPDRS (unified Parkinson’s disease rating scale), D-CNN (dynamic convolutional neural network), S-CNN (state transition probability convolutional neural network). The bold font in the table represents the machine learning algorithm with the highest accuracy.

## Data Availability

Not applicable.
